# Supplementary material for: Evidence for the Involvement of Vernalization-related Genes in the Regulation of Cold-induced Ripening in ‘D’Anjou’ and ‘Bartlett’ Pear Fruit
Source: Sci Rep. 2020 May 21;10:8478. doi: 10.1038/s41598-020-65275-8 (PMC7242362; doi:10.1038/s41598-020-65275-8)
Supplement: Supplementary file 4 — Supplementary information 4. [file 41598_2020_65275_MOESM4_ESM.pdf]

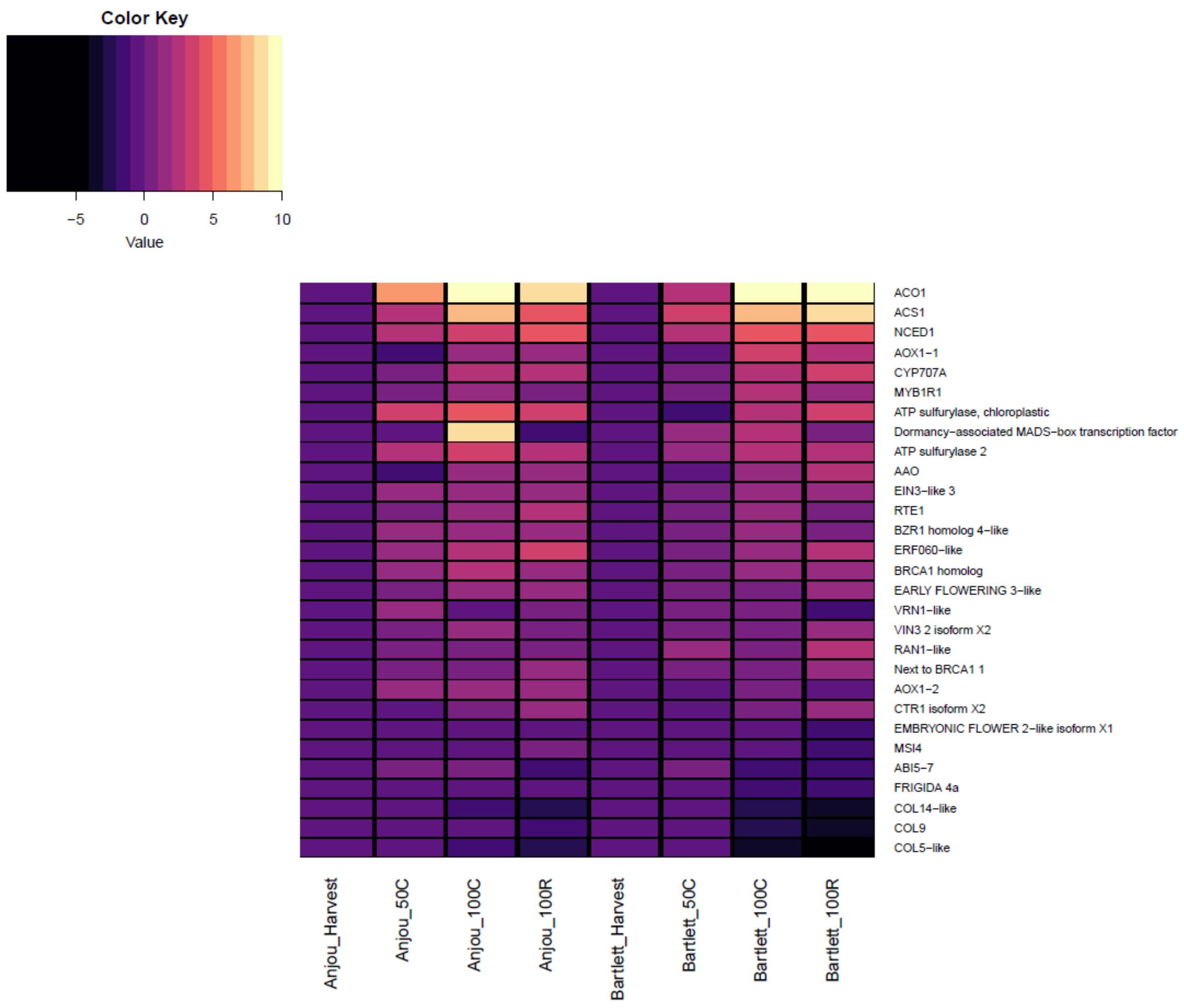

Supplementary Figure 4.1 Heatmap displaying Log_2 fold change expression of conditioned 'D'Anjou' and 'Bartlett' pear fruit contigs discussed in the manuscript. Expression values at Harvest for each genotype were used as the baseline for comparison. Heatmap was generated in R using the "gplots" package.

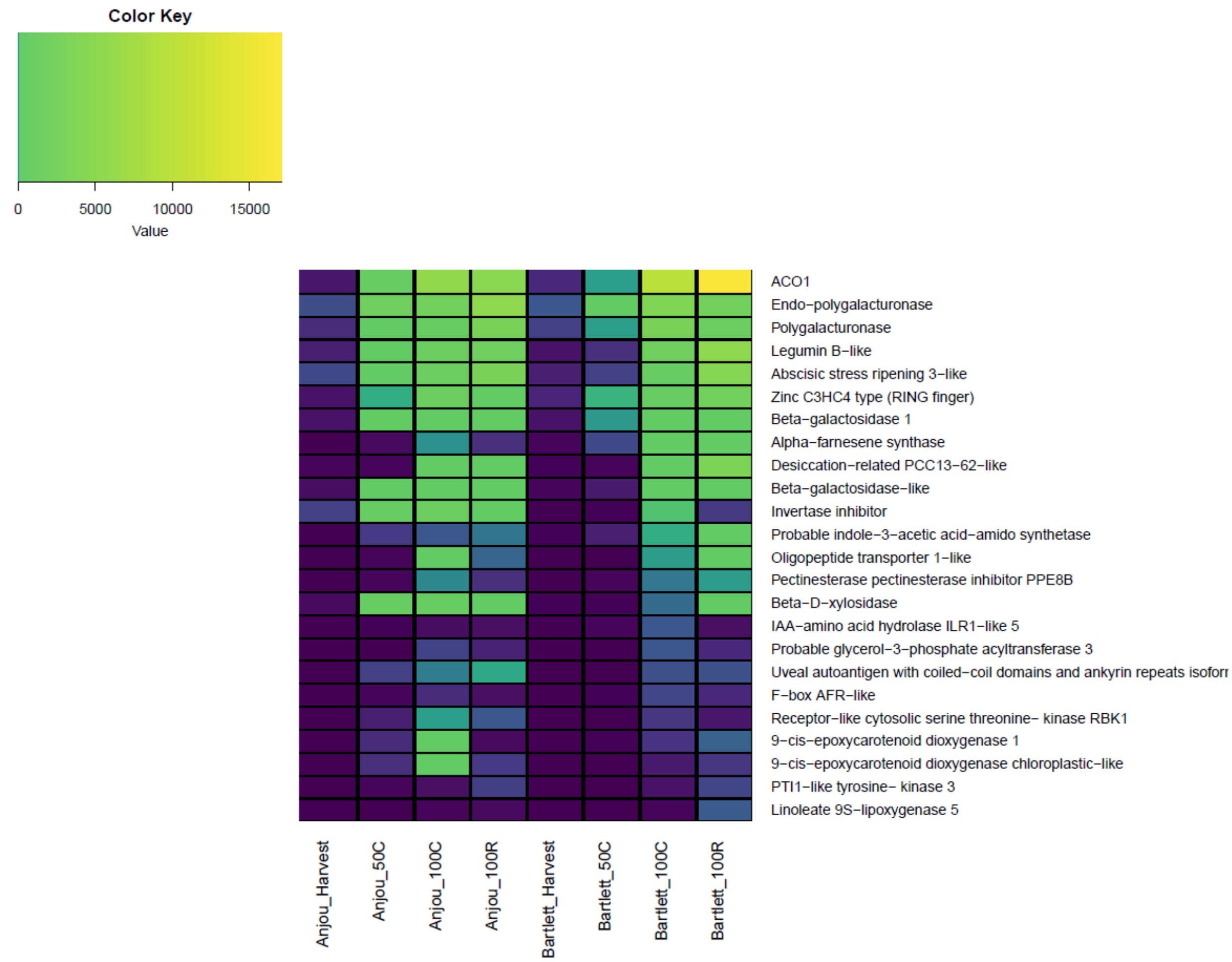

Supplementary Figure 4.2 Heatmap representing normalized expression (RPKM) values of 25 shared ‘D’Anjou’ and ‘Bartlett’ contigs with highest Log_2 fold change (FC) expression at the 100C time point. Expression values at Harvest for each genotype were used as the baseline for determining FC. Contigs were sorted by FC to generate lists of contigs with the highest FC expression changes from harvest to 100C. The ‘Venn Diagram’ application in OmicsBox was used to select shared contigs among both lists. The resulting list was filtered to exclude non-annotated contigs, as well as contigs with a p-value of greater than 0.05 (per the maSigPro time course differential expression analysis). Heatmap was generated in R using the “gplots” package.

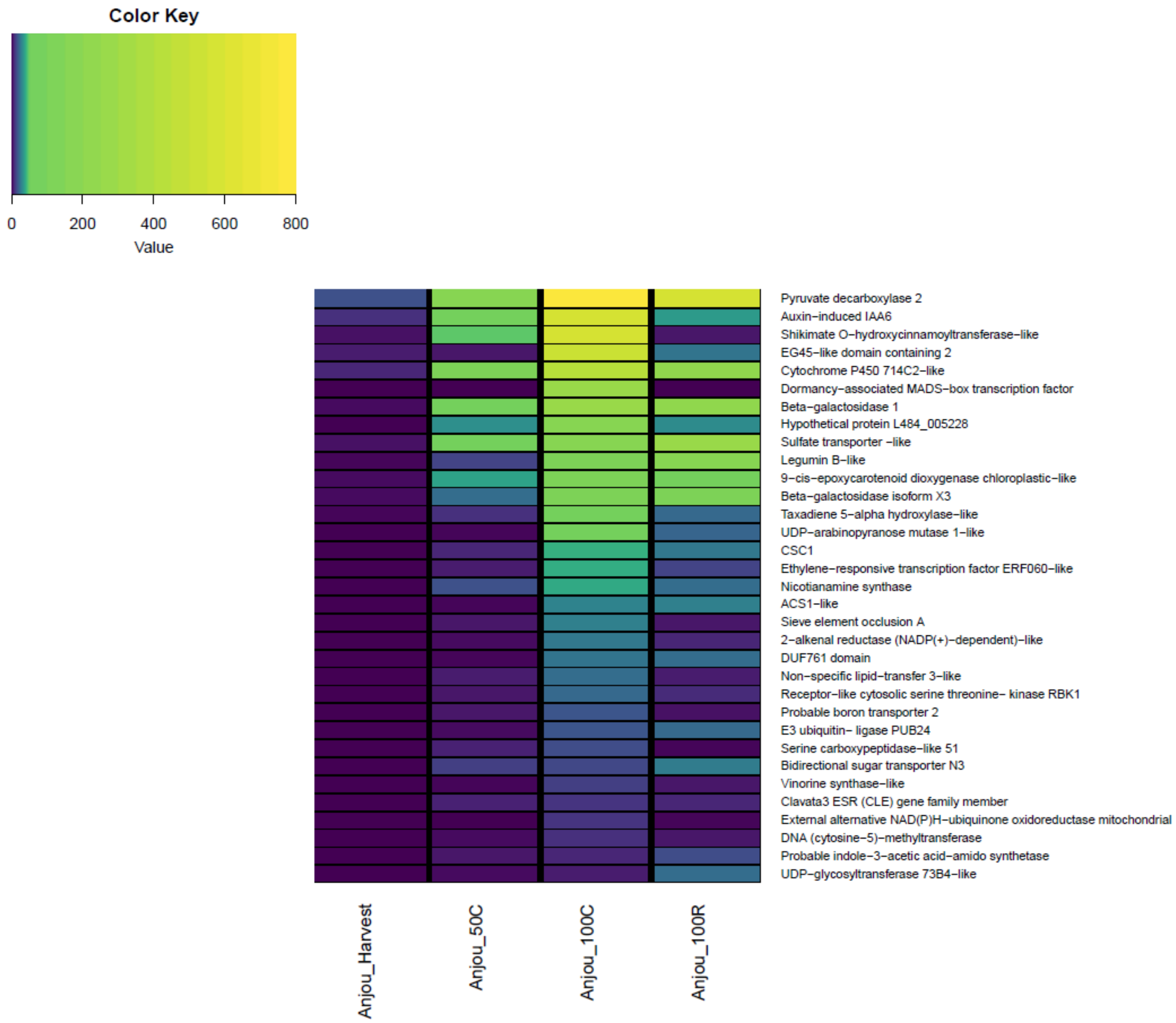

Supplementary Figure 4.3 Heatmap representing normalized expression (RPKM) values of 35 unique 'D'Anjou' contigs with highest Log_2 fold change (FC) expression at the 100C time point. Expression values at Harvest were used as the baseline for determining FC. Contigs were sorted by FC to generate lists of contigs with the highest FC expression changes from harvest to 100C. The 'Venn Diagram' application in OmicsBox was used to compare the list of top 100 genes with highest FC from each genotype, and filter for those unique to 'D'Anjou'. The resulting list was filtered to exclude non-annotated contigs, as well as contigs with a p-value of greater than 0.05 (per the maSigPro time course differential expression analysis). Heatmap was generated in R using the "gplots" package.

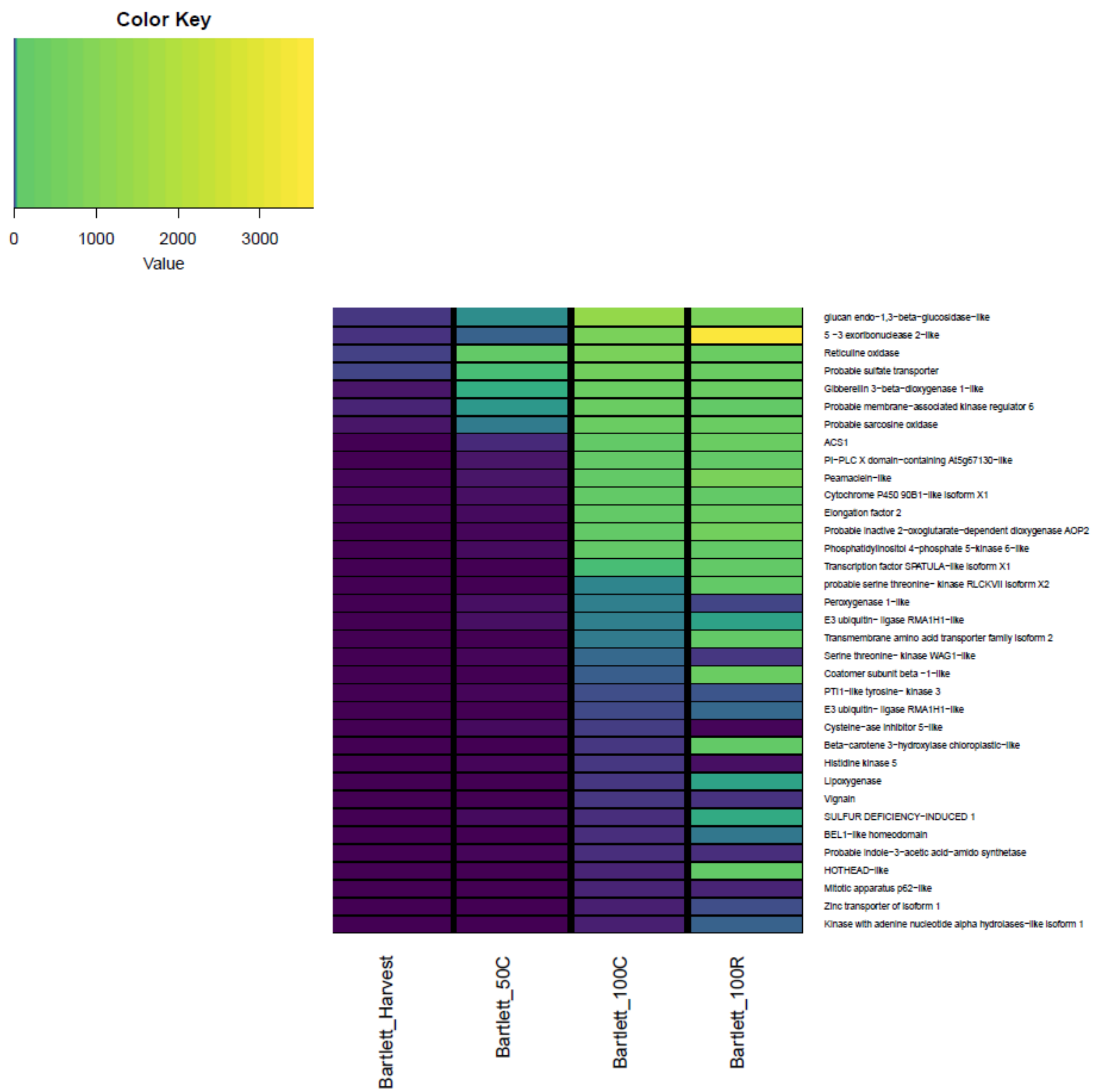

Supplementary Figure 4.4 Heatmap representing normalized expression (RPKM) values of 35 unique 'Bartlett' contigs with highest Log_2 fold change (FC) expression at the 100C time point. Expression values at Harvest were used as the baseline for determining FC. Contigs were sorted by FC to generate lists of contigs with the highest FC expression changes from harvest to 100C. The 'Venn Diagram' application in OmicsBox was used to compare the list of top 100 genes with highest FC from each genotype, and filter for those unique to 'Bartlett'. The resulting list was filtered to exclude non-annotated contigs, as well as contigs with a p-value of greater than 0.05 (per the maSigPro time course differential expression analysis). Heatmap was generated in R using the "gplots" package.

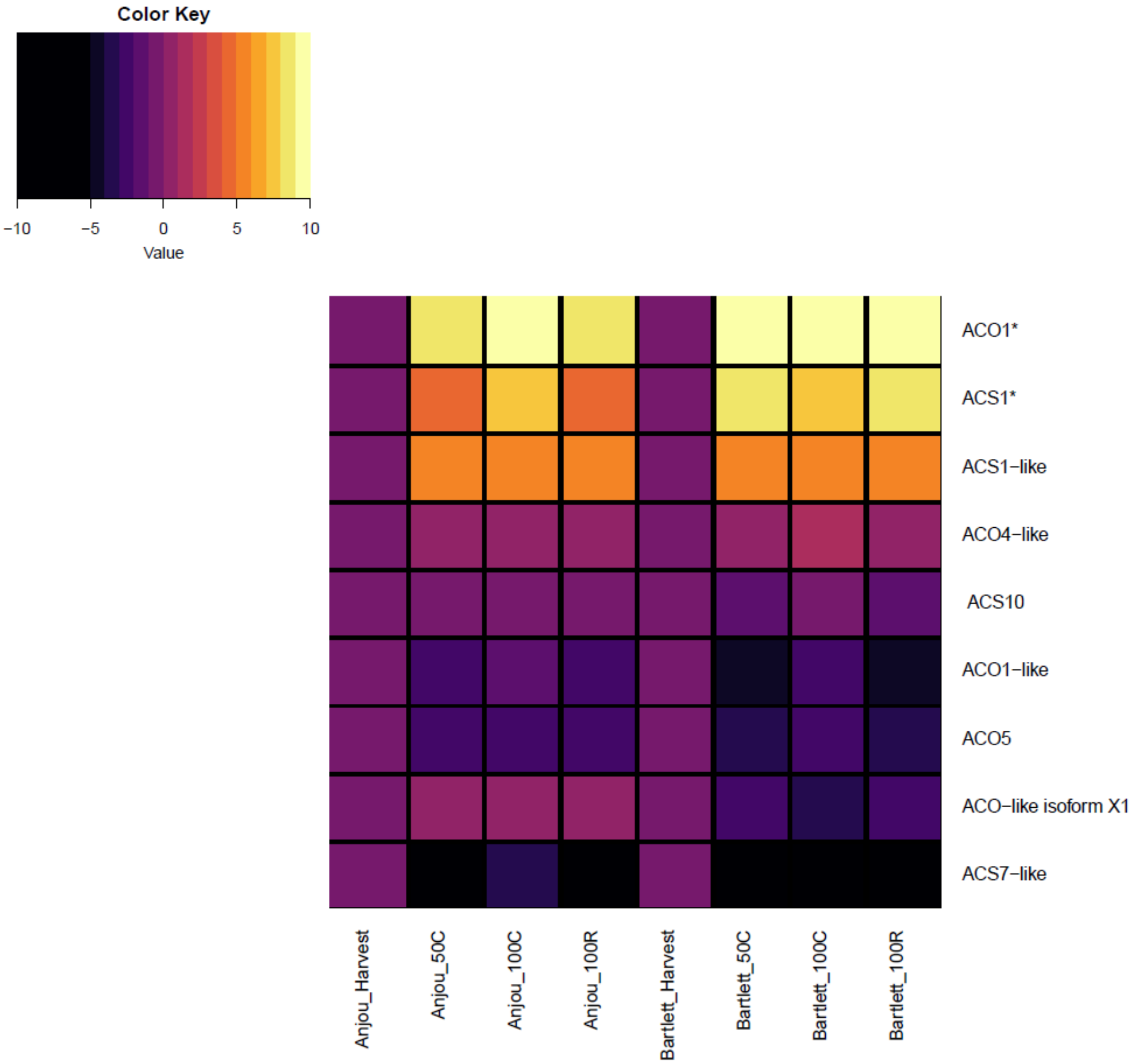

Supplementary File 4.5 Heatmap displaying Log_2 fold change (FC) expression of differentially expressed ACO and ACS isoforms. Expression values at Harvest for each genotype were used as the baseline for comparison. Heatmap was generated in R using the “gplots” package. Asterisks indicate isoforms discussed in the manuscript.
