# Supplementary material for: Evidence for the Involvement of Vernalization-related Genes in the Regulation of Cold-induced Ripening in ‘D’Anjou’ and ‘Bartlett’ Pear Fruit
Source: Sci Rep. 2020 May 21;10:8478. doi: 10.1038/s41598-020-65275-8 (PMC7242362; doi:10.1038/s41598-020-65275-8)
Supplement: Supplementary file 12 — Supplementary information 12. [file 41598_2020_65275_MOESM12_ESM.docx]

**Evidence for the Involvement of Vernalization-related Genes in the Regulation of Cold-induced Ripening in 'D'Anjou' and 'Bartlett' Pear Fruit**

Seanna Hewitt^1,3^, Christopher A. Hendrickson^2^, and Amit Dhingra^1,3*^

1 Molecular Plant Sciences, Washington State University, Pullman, Washington

2 National University, La Jolla, California

3 Department of Horticulture, Washington State University, Pullman, Washington

*Corresponding author: [adhingra@wsu.edu](mailto:adhingra@wsu.edu)

SUPPLEMENTARY FILES

**Supplementary File 1.** Firmness values for conditioned and non-conditioned ‘D’Anjou’ and ‘Bartlett’ pear fruit at each sampling time point.

**Supplementary File 2.** Annotated master assembly fasta for cold conditioned ‘D’Anjou’ and ‘Bartlett’ pear fruit.

**Supplementary File 3.** Mean RPKM values, standard error, and time course differential expression information for cold conditioned ‘D’Anjou’ and ‘Bartlett’ pear fruit.

**Supplementary File 4.** Heatmap representations of 1.) differentially expressed genes shown in Table 1, 2.) shared contigs with highest log fold change expression difference between ‘100% Conditioned’ and ‘Harvest’ time points, 3.) genotype-specific contigs with highest log fold change expression between ‘100% Conditioned’ and ‘Harvest’ time points, and 4.) log fold change expression of differentially expressed ACO and ACS homologs

**Supplementary File 5.** Differential expression graphs for of ethylene regulatory genes *BZR1* and *MSI4*

**Supplementary File 6.** Differential expression graphs for sulfur metabolism-associated genes.

**Supplementary File 7.** Differential expression graph for *Polycomb group embryonic flower 2-like isoform x1* (*EMF2*).

**Supplementary File 8.** Differential expression graphs for BRCA1 and Next-to-BRCA1 genes.

**Supplementary File 9.** All shared and unique enriched gene ontologies for cold conditioned ‘D’Anjou’ and ‘Bartlett’ pear fruit.

**Supplementary File 10.** RPKM data files and experimental design files used for MaSigPro time-series differential expression analysis in OmicsBox.

**Supplementary File 11.** Quantitative RT-PCR validation with calculated expression values.
